# Electroconductive hydrogel as an anode for the *Arthrospira platensis* based photobiofuel cell

**DOI:** 10.1186/s11671-026-04855-5

**Published:** 2026-08-20

**Authors:** Maria Aksenova, Vera Anisimova, Maria Vishnevskaya, Yulia Parunova, Daniil Sukhinov, Yana Sergeeva, Anna Baldycheva, Andrey Somov, Pavel Gotovtsev

**Affiliations:** 1 Moscow Institute of Physics and Technology (National Research University), 9 Institutskiy Per., Dolgoprudny, Moscow Region Russian Federation 141701; 2 Institute for Information, Transmission Problems of RAS, Moscow, Russian Federation; 3 Bauman Moscow State Technical University, Moscow, Russian Federation; 4 National Research Centre “Kurchatov Institute”, Akademika Kurchatova Pl., 1, Moscow, Russia 123182; 5 University of Exeter, Exeter, EX4 4QF UK; 6 Central University, Moscow, Russia 123056; 7 Research Center of Biotechnology of the Russian Academy of Sciences, 33, Bld. 2. Leninsky Ave., Moscow, Russia 119071; 8 Center for Engineering Systems and Sciences, Moscow, Russia 121205

**Keywords:** Photobiofule cell, PEDOT:PSS, *Arthrospira platensis*, Electrode, Hydrogel, Potassium hexacyanoferrate(III) trihydrate

## Abstract

Photobiofuel cells (PBFC) as ecological and self-sustainable energy sources are essential in a variety of monitoring applications. To put this technology in practice there is a number cells aspects to be improved, and one of them is electrodes material. Biocompatibility, conductivity and large surface are between the most reliable properties of such materials. In this article, we report on the development of a laboratory photobiofuel cell with new electrode materials based on an electrically conductive hydrogel with poly(3,4-ethylenedioxythiophene)-poly(styrenesulfonate) (PEDOT:PSS). In the course of the research work, the values of current and power density of 3.1 ± 0.2 µA/cm^2^ and 7.6 ± 0.2 µW/cm^2^ were achieved, respectively, by optimizing the parameters of the biomass used and adding a mediator. Also, we demonstrate that the addition of multi-walled nanotubes enhances the hydrogel conductivity, whereas the immobilization of cyanobacteria diminishes it.

## Introduction

Today, the main energy sources are fossil fuels, which have limited supply and are the major contributors to atmospheric CO_2_. That is why the development of renewable carbon–neutral and carbon-negative sources of energy is an essential problem [1].

An alternative source of energy is solar radiation collected by solar panels; however, they have disadvantages, such as toxic components contained in their composition [2] and the carbon footprint [3]. The promising green energy trend is the usage of dye-synsitized [4] and photobiofuel cells (PBFC) [5] In PBFC which the photosynthetic apparatus of microorganisms is used to produce electricity. Such cells can be used in variety of applications [5, 6], for example powering low-power wireless devices in the scope of Internet of Things [7, 8], wastewater treatment [9], and environmental sensors for water quality monitoring [10]. Also, microorganism’s based PBFS can fix CO_2_, that is unique ability to generate electricity and consume carbon dioxide. Despite all the advantages, this technology has a significant limitation in the form of low performance. The development of new materials can help overcome these limitations and increase the efficiency of PBFC. The working electrode must exhibit biocompatibility, electrochemical stability within the required potential range, and non-interaction with the surrounding environment. Additionally, it should possess electrical conductivity and a high specific surface area conducive to cell adhesion. Taking into account the prospective scalability of the technology, materials with reasonable cost are preferable.

Three generations of utilized anodes (working electrodes) can be distinguished [11]. In the first-generation PBFCs, flat metal plates were used [12, 13], which were readily available but had several significant drawbacks, making them highly ineffective for use in such devices. The flat surface did not promote cell adhesion and provided a small effective surface area.

Since the 2000s, carbon in its various forms has been the most attractive material for second-generation PBFC’s electrodes. Carbon fabric, in particular, is commonly used, but it is hydrophobic and has low electrical conductivity, so it is subjected to modifications [14, 15]. For example, to increase conductivity, it is coated with a conductive composite polymer, poly(3,4-ethylenedioxythiophene)-poly(styrenesulfonate) (PEDOT:PSS), and for hydrophilicity, with polytetrafluoroethylene [14]. Carbon nanotubes [16], graphite [17], thin ITO/FTO films on substrates [18, 19] are also used. Carbon paint mixed with the conductive polymer polypyrrole is also encountered [20]. Carbon materials remain a common choice for working electrodes [21], but they are not without criticism due to their lower conductivity compared to metals [22].

The third generation of working anodes differs from others in having a highly porous bulk structure, which provides the greatest effective surface area. The most commonly used material is ceramics with various conductive fillers and coatings [23–25]. These materials are characterized by high light transmission, hydrophilicity, conductivity, versatility, nano-roughness, and ease of tailoring. At the same time, they have some disadvantages, e.g. moderate cost and limited electrochemical window. Another approach in conductive polymers based structured materials as an electrode [41, 42]. The conductivity of PEDOT:PSS and its derivatives is lower in comparison with metals and carbon materials [43], but possibility to build structures with surface landscape or/and porous characteristic size commensurate to the microorganism’s dimensions [44]. Thus, distance between microorganism and material became lower that leads to more efficient electron transfer in general [45, 46].

Cyanobacteria are of particular interest for use in biotechnology. They possess a distinctive feature: thylakoids are not located in chloroplasts but rather in the cytoplasm of cells. On the outer surface of thylakoid membranes, light-capturing pigment-protein complexes called phycobilisomes are situated, incorporating phycobiliproteins such as phycocyanin, allophycocyanin, and phycoerythrin. The assumed positioning of thylakoids, where electrogenic reactions take place in their membranes, facilitates the electron transfer from the photosynthetic chain to the anode. However, the precise mechanism of this process remains unclear [5].

The cultivation of cyanobacteria on the anode can be either axenic or non-axenic. Among pure cultures, *Synechocystis* sp. PCC 6803 [14, 20, 26–29], *Synechococcus elongatus* [30], and *Anabena* sp. [30] are most commonly employed. A distinctive feature of *Synechocystis* sp. PCC 6803 is its ability to form electrically conductive nanowires when cultivated under conditions of carbon dioxide limitation and excess light. Thus, *Synechocystis* sp. can be utilized as a mediator between heterotrophic microorganisms and the anode or as a standalone pure culture on the anode [31]. Additionally, successful studies have been conducted using *Arthrospira platensis* [32]. Variants of PBFCs with microalgae *Parachlorella* spp. and *Chlorella vulgaris* have also been reported [19]. Among the strains of these species, both freshwater and marine, a comparison revealed a higher power density when using the former in PBFCs, while the latter exhibited better current density stability [19].

The properties of the utilized biomass significantly impact the power output of PBFC. For instance, in a study [28], the influence of the *Synechocystis* sp. PCC 6803 biomass concentration on the anode was analyzed. Environmental conditions, such as temperature and light exposure, also play a role in affecting phototrophic microorganisms [31], thereby influencing PBFC performance.

Experimental studies have demonstrated that the power output density can reach 50 µW/cm^2^, but typically, this value does not exceed 10 µW/cm^2^ [34]. As discussed earlier, the primary avenues for enhancing efficiency of these devices involve the development of new electrodes and improving the efficiency of charge transport from the biological component to the electrode. To increase efficiency of charge transport the novel materials for electrodes should be found. Thus, novelty of this research is in the application of electroconductive hydrogel as electrode material and mediator-based electron transport study.

This article is organized as follows: in Sect. 2 we present material and methods used in this research. In Sect. 3 we demonstrate results achieved in this work. Finally, we provide concluding remarks and discuss our future work in Sect. 4.

## Materials and methods

### Biomass cultivation

As the anodic biological material, the strain of the cyanobacterium *Arthrospira platensis* B-12619 from the All-Russian Collection of Industrial Microorganisms was selected. Cultivation was carried out in modified Zarrouk medium [35] in 50 ml Erlenmeyer flasks containing 25 ml of suspension. The cultivation process took place in a shaking thermostat, specifically the Eppendorf New Brunswick Innova 42/42R, with continuous stirring at 140 rpm, illumination at 14–16 μmol/(m^2^ s), and a temperature of 30 °C. Inoculation involved the use of a 4-day *Arthrospira platensis* suspension, and the initial OD_750_ value was adjusted to 0.05.

The growth processes were monitored by determining the optical density of the culture at 750 nm using a ThermoScientific Genesys 10S UV–Vis spectrophotometer, USA.

### Determination of C-phycocyanin content

During sample collection, a suspension with a volume of V (ml) was taken, and then the biomass was precipitated by centrifugation (7500 rpm, 15 min) using the Apexlab XC-H16 centrifuge, rotor A011-3. The biomass was washed three times with distilled water to remove adsorbed salts.

C-Phycocyanin (C-PC) extraction was carried out through a comprehensive freezing/thawing cycle (− 22/ + 25 °C) performed three times. Sodium phosphate buffer (pH 7.0 was) used as the extractant. Cellular debris separation was achieved by centrifugation (12,500 rpm, 30 min) [36]. The C-PC concentration (1) was calculated according [37] and C-PC content was calculated using formula (2).

1 $$ \left[ {{\mathrm{C}} - {\mathrm{PC}}\left( {\frac{{{\mathrm{mg}}}}{ml}} \right)} \right] = \frac{{{\mathrm{A}}_{620} - 0,474 \times {\mathrm{A}}_{650} }}{5,34} $$

2 $$ \left[ {C - {\mathrm{PC}}\left( \frac{mg}{g} \right)} \right] = \frac{{\left[ {{\mathrm{PC}}\left( {\frac{mg}{{ml}}} \right)} \right] \times V_{sol} \left( {ml} \right)}}{{m_{DCW} \left( g \right)}} $$

A_λ_ – optical density at λ nm, *V*_*sol.*_ – volume of extracted phycocyanin solution, m_DCW_ – Dry cell weight of biomass contained in a volume V of suspension.

The dry weight of biomass was determined by lyophilization. A suspension with a volume of V was sampled, and the biomass was precipitated by centrifugation (7500 rpm, 15 min), followed by three washes with distilled water. The precipitated biomass was frozen at − 70 °C in a CryoCube F101h -86 low-temperature freezer, then subjected to the lyophilization process in a Labconco FreeZone 2.5 L sublimation dryer.

### Electrochemical and electrophysical methods

For a comparative analysis of the photocurrent, cyclic voltammetry was conducted under various conditions (in light and darkness, with a cell suspension on the anode, and with an anode impregnated only with the medium without cells) using a Metrohm Autolab PGSTAT204 potentiostat with a scan rate of 5 mV/s in a voltage range from − 0.3 to + 0.3 V with a step of 2.44 mV. Chronoamperometry with a step of 0.5 s was employed to determine the steady-state current.

The hydrogel samples were investigated using electrochemical impedance spectroscopy with an Autolab PGSTAT302N potentiostat/galvanostat in the frequency range from 10 kHz to 0.1 Hz. Before measurements, the gels were swollen for 50 min in Zarouk medium, and the measurements were conducted in an electrochemical cell in Zarouk medium. A three-electrode cell was used, with the studied gel as the working electrode, a silver chloride electrode as the reference electrode, and a platinum electrode as the auxiliary electrode.

Electrical conductivity of dry electrode was measured using Keithley 4200 SCM station. Van der Pauw standard four-probe setup was applied according to [38]. All samples were dry and have next dimensions: 15 × 15 mm, 100 mkm thickness. Three samples of dry gels with nanotubes and three without were used for the measurements.

### Electrode preparation

Anode: The working electrode was a hydrogel prepared using the method outlined in [36] with the addition of carbon nanotubes. Solutions of polyvinyl alcohol (PVA) and carrageenan (CRG) with a concentration of 1.3% were separately prepared at 70 °C. Subsequently, these two components were mixed with 2.6% PEDOT:PSS in a volume ratio of 1:1:2, also maintained at a constant temperature of 70 °C. This resulted in a mass ratio of components in the mixture of 1:1:1. At this stage, 2% by weight of multiwall carbon nanotubes modified with polyaniline were added, with a mass fraction of carbon nanotubes at 65% and a mass fraction of polyaniline at 35% (“Taunit-M”, Russia). The mixture then underwent 4 cycles (each lasting 24 h) of freezing and thawing (− 12/ + 25 °C) in a Petri dish. In the final stage of gel preparation, it was dried at 40 °C to complete dryness. The dimensions of the hydrogel electrode used in the PBFC were 15 × 15 mm in dry state. For the electrochemical studies of the material, a similar hydrogel was obtained, differing only in the absence of nanotubes. All specific characteristics current density and power density were applied to the macro surface area of anode in the dry state. Stady-state current was used to determine those characteristics.

Cathode: A nickel plate with a working area of 1.1 cm^2^ served as the cathode.

### Photobiofuel cell assembly

**Fig. 1:**
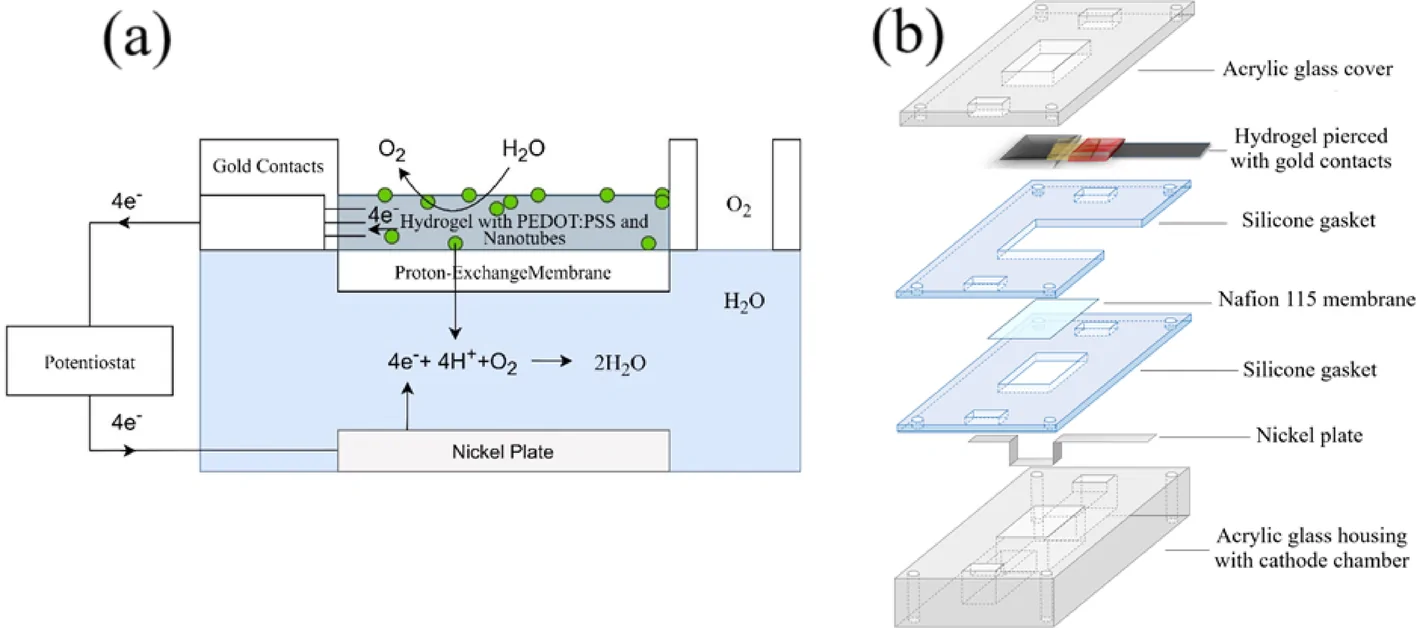
**a** Photobiofuel cell scheme, **b** 3D model of cell design

The schematic diagram of the Photobiological Fuel Cell (PBFC) is illustrated in Fig. 1.

The PBFC housing was constructed from acrylic glass. The upper, anodic chamber contained a working electrode based on a hydrogel with the conductive polymer PEDOT:PSS and carbon nanotubes, onto which a suspension of *A. platensis* cells was applied. This chamber had free access to air and light. Gilded pins, penetrating the hydrogel, served as the contact to connect the anode with the external electrical circuit. The lower, cathodic chamber housed a nickel plate and was filled with water, allowing air access. The cathodic and anodic chambers were separated by a proton-conductive Nafion 115 membrane (Sigma-Aldrich, USA). Before being applied to the anode, biomass was precipitated by centrifugation (7500 rpm, 15 min).

The anode was illuminated by three LEDs of white, red, and blue colors with the following specifications: power supply 11.8 V, power 1.9 W, collectively providing illumination of 70–130 µmol/(m^2^ s), measured using ALMEMO 2450-1R02.

At first basic characteristics of PBFC were determined with microorganisms in the stationary growth phase. Next influence of C-PC content was determined and finally PBFC was investigated with microorganisms with optimal C-PC content. The final experiments were provided by the next sequence: cell-free medium, PBFC with microorganisms without mediator and without illumination, PBFC with microorganisms and with illumination and PBFC with illumination and with mediator. All electrochemical measurements were provided in two electrode setup according [47, 48] to emulate real-world performance of PBFC.

### Mediator

Two experiments were conducted with the addition of a mediator to the anode chamber. Using 13-day-old biomass, potassium ferricyanide trihydrate was added at a concentration of 0.01 mmol/l per volume of the anode chamber; the concentration of a stock aqueous solution is 0.1 mmol/l.

### Electron microscopy study

To investigate anode morphology scanning electron microscopy in environmental scanning mode was provided. Versa 3D DualBeam (SEM, FEI, USA) was used in this study.

## Results

### The positioning of *Arthrospira platensis* on the anode

The anode was illuminated by three LEDs of white, red, and blue colors with the following specifications: power supply 11.8 V, power 1.9 W, collectively providing illumination of 70–130 μmol/(m^2^ s), measured using ALMEMO 2450-1R02.

**Fig. 2:**
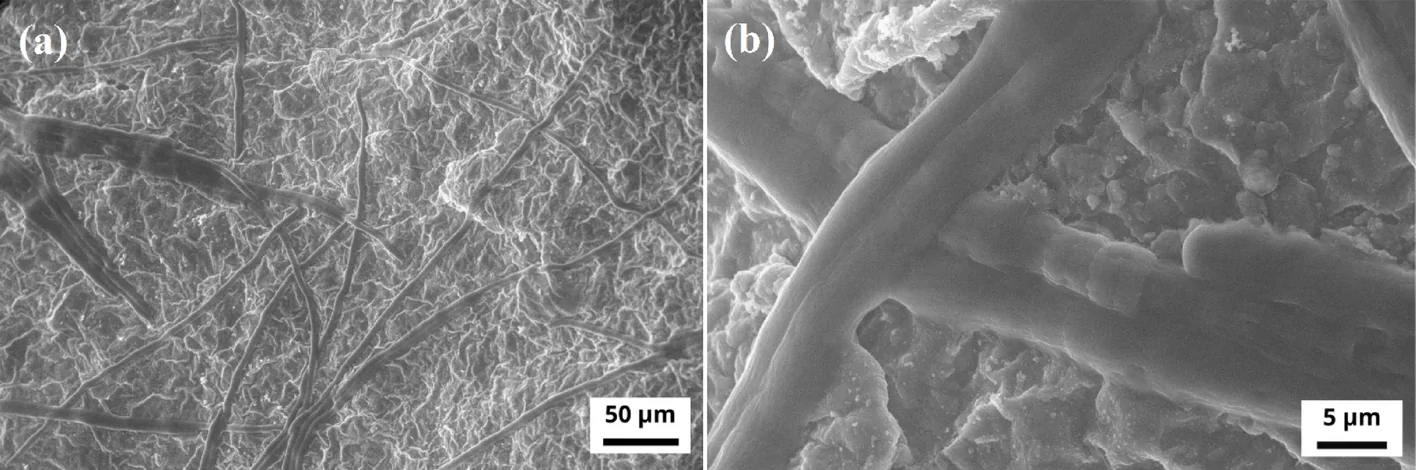
*Arthrospira platensis* trichome on a hydrogel **a** magnification 500x, **b** magnification 4000x

An image of the trichome of the cyanobacterium *Arthrospira platensis*, applied to a hydrogel (anode), was obtained using scanning electron microscopy (SEM). Presented in the Fig. 2 images reveal the interaction between the cells and the hydrogel. The trihomes are closely placed to the hydrogel surface that can positively influence on the efficiency of the electron transfer from cells to the electrode material.

### Impedance spectrometry of the hydrogels

**Fig. 3:**
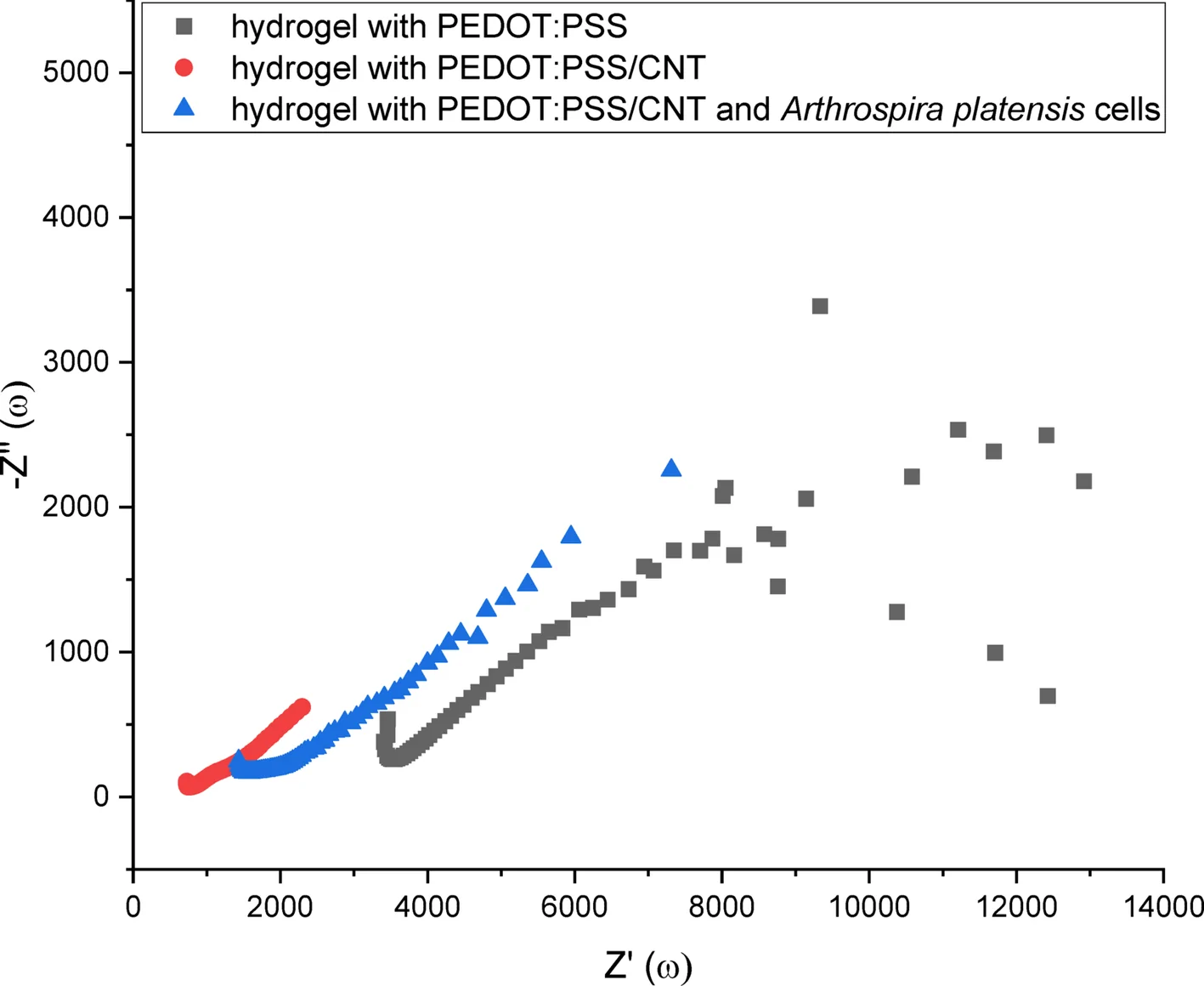
Nyquist diagrams for hydrogels. CNT – carbon nanotubes

Figure 3 presents Nyquist diagrams for three hydrogels: two containing multi-walled carbon nanotubes: one without *A. platensis* cells, one with immobilized *A. platensis* cells, and one without cells and nanotubes. The diagram shows that the addition of nanotubes to the hydrogel increases its conductivity, but the immobilization of cyanobacteria reduces it. This latter effect may be related to the influence of microorganisms on the material's structure in the immobilization region. It was previously shown [38] that the electrophysical properties of hydrogels change during their swelling process, which involves structural rearrangement. As seen from the SEM results (Fig. 2), *A. platensis* trichomes are in close contact with the hydrogel. It is known that these cyanobacteria actively synthesize and secrete various polysaccharides [39], which are likely to interact with the hydrogel and affect the material's structure and swelling, and consequently its conductivity.

Increasing conductivity due to nanotubes adding also shown by dry gels resistivity measurements. The resistivity of electrodes without nanotubes was equal to 25–28 kOhm for the sample, that is corresponds with our previous study [38]. Nanotubes additions provide lower resistivity equal to 8–12 kOhm for the sample.

### Obtaining basic characteristics of the PBFC

**Fig. 4:**
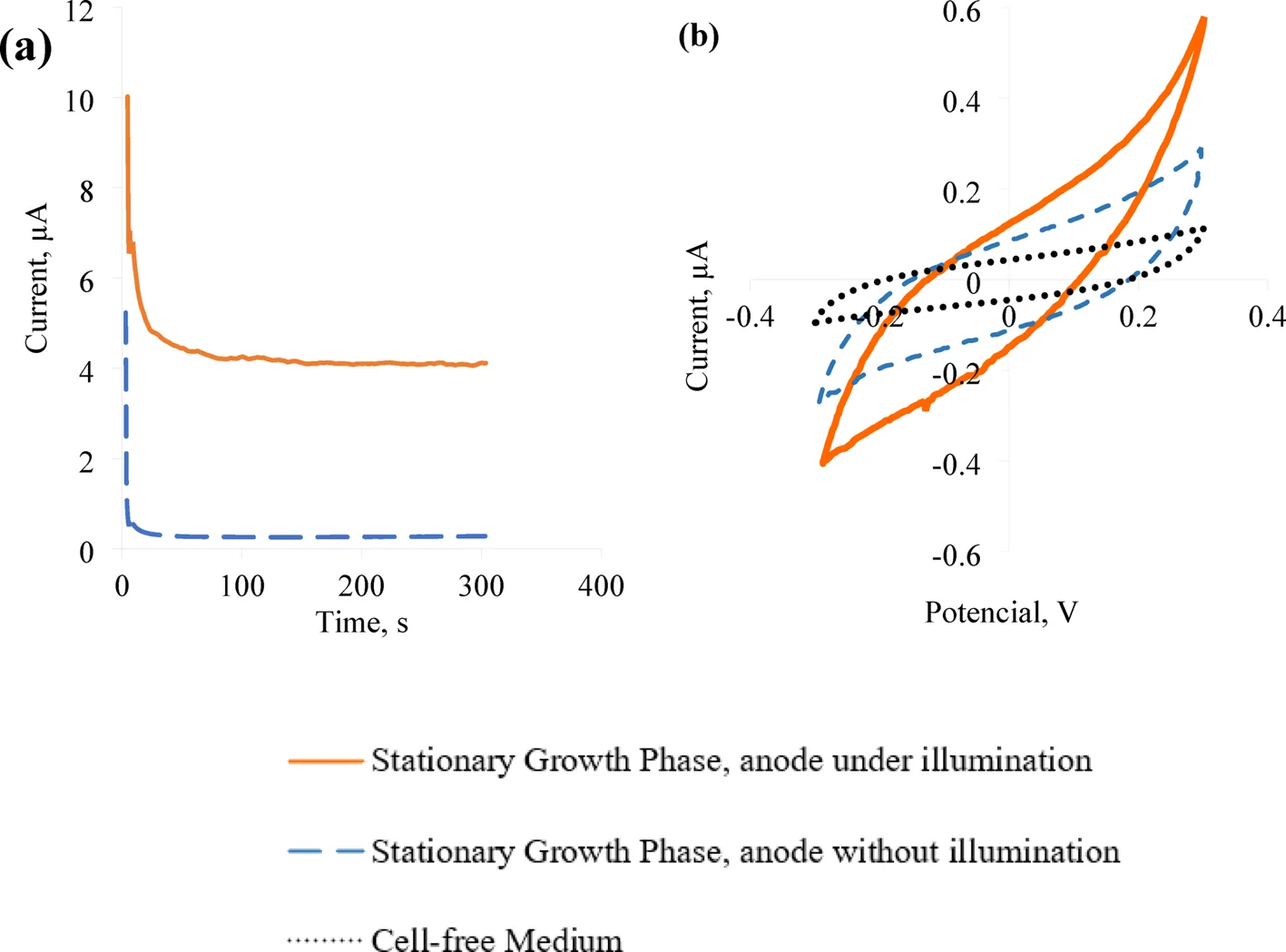
**a** Chronoamperometry and **b** CVs of the PBFC with Biomass in the Stationary Growth Phase under Illuminated and Dark Anode Conditions

To establish the fundamental characteristics of the PBFC, biomass in the stationary growth phase was utilized. Chronoamperometry was employed, revealing a consistent current of 4.0 ± 0.2 µA under anode illumination Fig. 4a, cell-free medium line is not presented, because current is lesser than 0,05 µA and cannot be visible in the chosen scale of current axis.

To ensure that the current was facilitated by the vitality of cyanobacteria, a comparison of cyclic voltampere characteristics (CVs) was conducted (Fig. 4b):

1. PBFC with an illuminated anode onto which a cell suspension was applied (1 ml with 10 mg dry weight of biomass).
2. PBFC with a non-illuminated anode onto which a cell suspension was applied (1 ml with 10 mg dry weight of biomass).
3. Control: PBFC with an anode onto which only a nutrient medium without cyanobacteria was applied (1 ml).

The difference in currents between the first and second experiments indicates the presence of a photocurrent. Additionally, a discrepancy between the second and third cases suggests the existence of a current facilitated by the respiratory electron transport chain.

### Dependency of PBFC performance on phycocyanin content in biomass

**Fig. 5:**
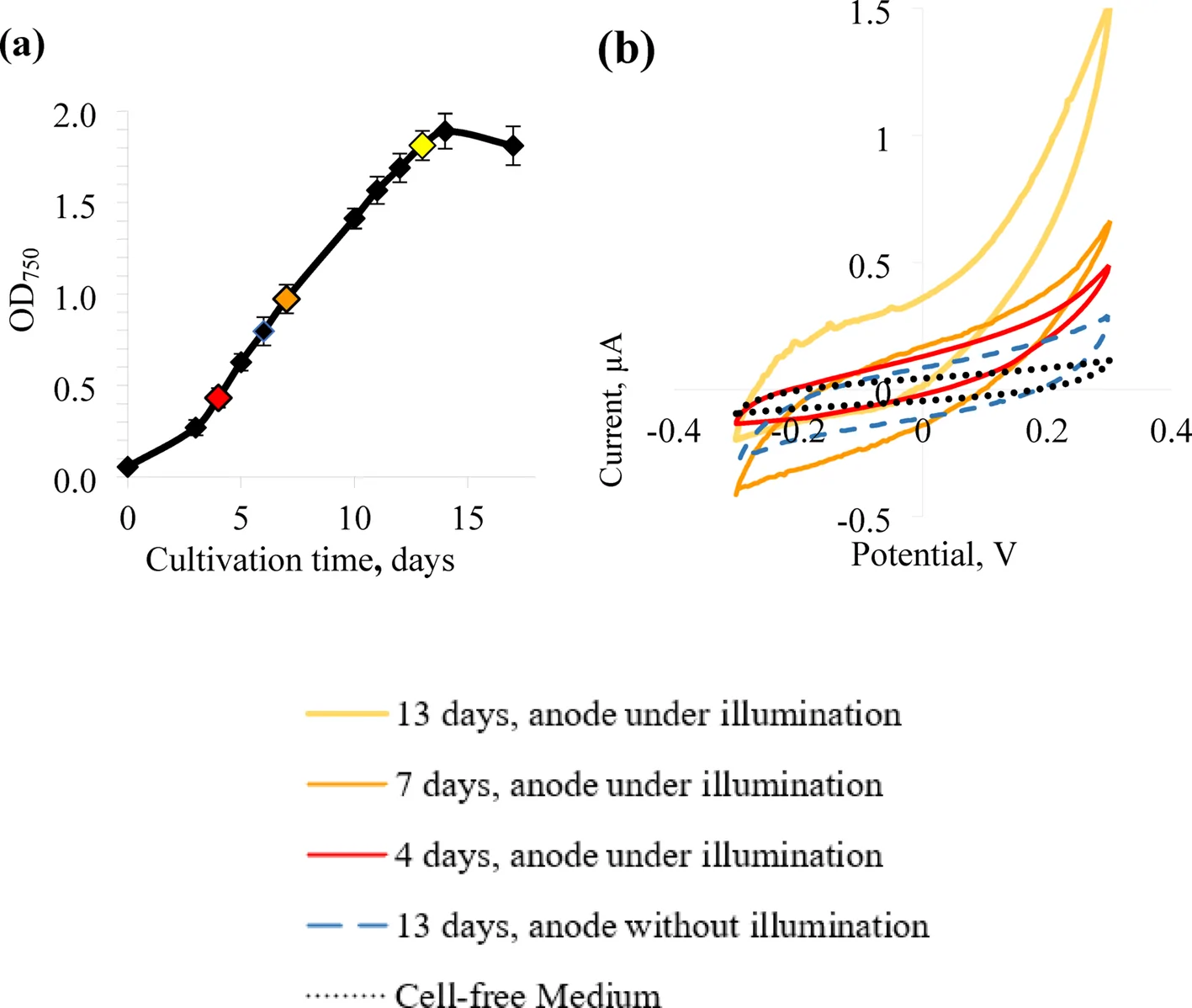
Influence of biomass age and electrochemical performance of biofuel cell. **a** Growth Curve of Cyanobacterium *A. platensis* OD_750_. **b** CVs Depending on the Age of the Utilized Biomass

To determine the optimal age of biomass and its phycocyanin content for use in the PBFC, an experiment was conducted, during which biomass was sampled on days 4, 7, and 13, corresponding to the beginning, middle, and end of the linear growth phase (Fig. 5).

An analysis of phycocyanin content was also conducted, and the results are presented in Table 1.

**Table 1:** Phycocyanin Content in *A. platensis* Biomass

Biomass age, days	Phycocyanin content, %
4	11.8 ± 1.4
7	14.8 ± 0.8
13	18.8 ± 0.4

Additionally, cell suspensions were sampled for use in the PBFC on days 4, 7, and 13 (10 mg dry weight of biomass), and cyclic volt ampere characteristics (CVs) were obtained (Fig. 5b). A direct correlation between the photocurrent and the phycocyanin content in the biomass was observed.

### Utilization of mediator-assisted electron transfer

**Fig. 6:**
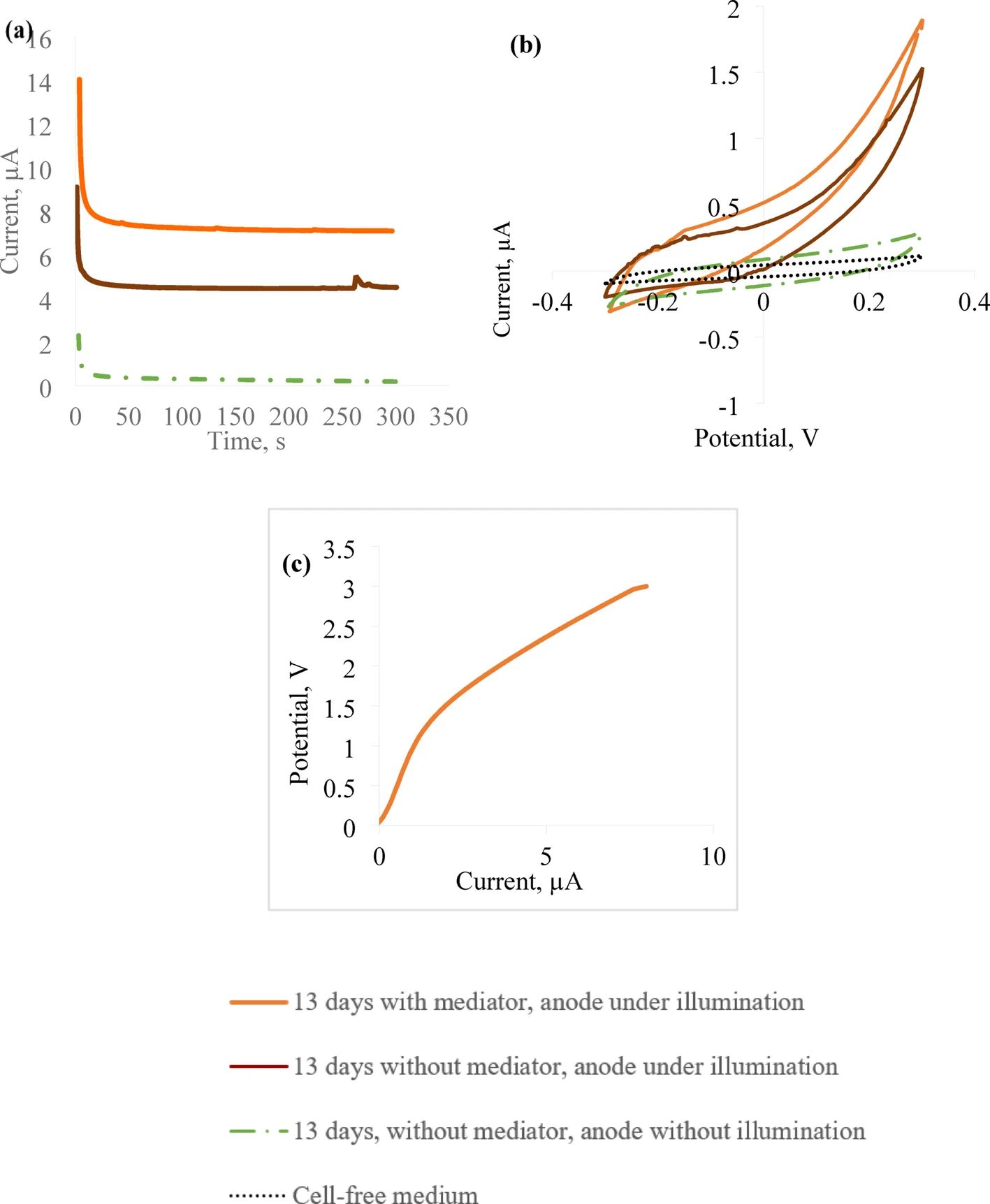
**a** Chronoamperometry, **b** CVs of the PBFC with Biomass at the End of the linear growth phase with mediator and without mediator (potassium hexacyanoferrate(III) trihydrate), under illuminated and dark anode conditions, **c** linear Sweep Voltammetry of PBFC under optimal conditions

In the PBFC using 13-day-old biomass, a mediator, potassium hexacyanoferrate(III) trihydrate, was added to the suspension at a concentration of 0.1 mmol/L to facilitate electron transfer to the anode [40]. The comparisons of CVs and chronoamperometry with and without the addition of the potassium hexacyanoferrate(III) trihydrate are illustrated in Fig. 6a, b. Cell-free medium line is not presented in the Fig. 6a, because current is lesser than 0.05 µA and cannot be visible in the chosen scale of current axis.

Based on the chronoamperometry data, current densities were calculated with and without the potassium hexacyanoferrate(III) trihydrate. The current density increased from 2.0 to 3.1 µA/cm^2^.

Thus, for PBFC optimization, the use of a mediator and biomass at the end of the linear growth phase, characterized by the highest phycocyanin content of 18.8 ± 0.4%, is essential. Under these conditions, the current density reached 3.1 ± 0.2 µA/cm^2^. The open-circuit voltage with 13-day-old biomass and mediator was 290 ± 10 mV. The calculated via Linear Sweep Voltammetry (LSV) (Fig. 6c) power output of the PBFC was 7.6 ± 0.2 µW/cm^2^.

## Conclusions

In this research, we have addressed the problem of ecological and self-sustainable energy sources. A Photobiological Fuel Cell (PBFC) was developed with novel electrode materials. A conductive hydrogel based on PEDOT:PSS with the addition of nanotubes was synthesized for use as the anode. The investigation of hydrogel conductivity revealed that the addition of multi-walled nanotubes enhances its conductivity, while the immobilization of cyanobacteria reduces it. With the use of a mediator potassium hexacyanoferrate(III) trihydrate, the PBFC current density increased from 2.0 to 3.1 µA/cm^2^. Under optimal PBFC conditions, current density reached 3.1 ± 0.2 µA/cm^2^, open-circuit voltage was 290 ± 10 mV, and power output was 7.6 ± 0.2 µW/cm^2^.

These results provide a basis for further optimization and scaling of the developed PBFC with new electrode materials. Prospective research directions include long-term PBFC operation testing, increasing the electrode's working area, and studying biomass growth on the anode.

## Data Availability

Data sharing not applicable to this article as no datasets were generated or analysed during the current study.
